# The heat‐shock protein/chaperone network and multiple stress resistance

**DOI:** 10.1111/pbi.12659

**Published:** 2017-02-23

**Authors:** Pierre Jacob, Heribert Hirt, Abdelhafid Bendahmane

**Affiliations:** ^1^ Institute of Plant Science—Paris‐Saclay Orsay France; ^2^ Center for Desert Agriculture King Abdullah University of Science and Technology Thuwal Saudi Arabia

**Keywords:** multistress resistance, stress acclimation, HSPs/chaperones, HSF, crop improvement

## Abstract

Crop yield has been greatly enhanced during the last century. However, most elite cultivars are adapted to temperate climates and are not well suited to more stressful conditions. In the context of climate change, stress resistance is a major concern. To overcome these difficulties, scientists may help breeders by providing genetic markers associated with stress resistance. However, multistress resistance cannot be obtained from the simple addition of single stress resistance traits. In the field, stresses are unpredictable and several may occur at once. Consequently, the use of single stress resistance traits is often inadequate. Although it has been historically linked with the heat stress response, the heat‐shock protein (HSP)/chaperone network is a major component of multiple stress responses. Among the HSP/chaperone ‘client proteins’, many are primary metabolism enzymes and signal transduction components with essential roles for the proper functioning of a cell. HSPs/chaperones are controlled by the action of diverse heat‐shock factors, which are recruited under stress conditions. In this review, we give an overview of the regulation of the HSP/chaperone network with a focus on *Arabidopsis thaliana*. We illustrate the role of HSPs/chaperones in regulating diverse signalling pathways and discuss several basic principles that should be considered for engineering multiple stress resistance in crops through the HSP/chaperone network.

## Introduction

Stresses are defined as environmental constraints that differ from optimal conditions, ultimately impeding growth and development. As sessile organisms, plants are commonly exposed to fluctuating environments and can show a great degree of resilience to conditions that would be considered harmful to many other organisms. The process by which an organism reaches phenotypic stability despite environmental and genetic variations was termed ‘canalization’ (Waddington, 1961).

To improve canalization to extreme conditions, the selection of stress resistance traits has been aided by the use of associated genetic markers. Single stress resistance traits have been extensively introgressed into elite cultivars. Due to the difficulty in reproducing a specific stress of a specific strength, most genetic studies have remained limited to single stress resistance. In nature, however, stresses rarely come alone. For instance, heat stress is associated with high light, but also facilitates the spreading of pests and pathogens leading to dramatic production losses. Moreover, responses to heat will involve the opening of stomata to dampen the rise in temperature, whereas a response to drought requires the closure of stomata to avoid water loss. In this regard, it is not surprising that responses to multiple, co‐occurring stresses are dramatically different than single stress responses added together. Transcriptomic analyses lead to the astonishing finding that 61% of the genes induced by dual stresses were not induced by any of the single stresses (Rasmussen *et al*., 2013). The combination of single stress resistance traits will consequently mostly not lead to multiple stress resistance. It is absolutely necessary to study multistress resistance pathways to understand and enhance canalization in the field (for review see Mittler and Blumwald (2010); Suzuki *et al*. (2014)).

One way to study multistress pathways would be to take advantage of the pleiotropic HSP (heat‐shock protein)/chaperone network. By definition, protein denaturation is a constant direct or indirect consequence of any stress, as stresses are defined as factors impeding normal cellular functions carried out by proteins. Potentially, any stressor that induces protein misfolding would require HSP/chaperone recruitment. In this regard, chaperones are now considered as powerful buffers against environmental stress and even genetic variations (Carey *et al*., 2006). Protein misfolding is the main feature of heat stress, so the HSPs were the first chaperones to be studied. However, since the discovery of HSPs/chaperones, it has been found that the role of these factors is not limited to heat stress management but is also involved in other stresses, such as cold, osmotic, drought, salt, UV, high light, oxidative stress and pathogen infection (Swindell *et al*., 2007).

### Multistress resistance and the HSP/chaperone pathway

HSPs and chaperones are found in most prokaryotes and eukaryotes, and even some viruses (Maaroufi and Tanguay, 2013). In a cell, more than 10 000 proteins co‐exist in a limited space. Biochemists worldwide have experienced the difficulty in producing only a few of these proteins in a native conformation in concentrations comparable with *in vivo* conditions. Unfolded proteins tend to form large aggregates that severely impede normal cellular functions. The main function of HSPs/chaperones is to act as a buffer to limit misfolding and resolve aggregates. By doing so, they minimize the impact of environmental and genetic variations on the proteome. HSP90 alone makes up for 1%–2% of the total protein content in eukaryotes (Krukenberg *et al*., 2011). The molecular mechanisms underlying the functions of HSPs have been extensively reviewed (Al‐Whaibi, 2011; Fu, 2014; Niforou *et al*., 2014; Wang *et al*., 2013). Chaperone functions are not limited to folding and HSP70 and HSP90 and their cochaperones have clearly been linked to signalling, protein targeting and degradation (Huang *et al*., 2014; Kadota and Shirasu, 2012; Kriechbaumer *et al*., 2012; Lee *et al*., 2009).

### Transcriptional control of HSPs

**Figure 1:**
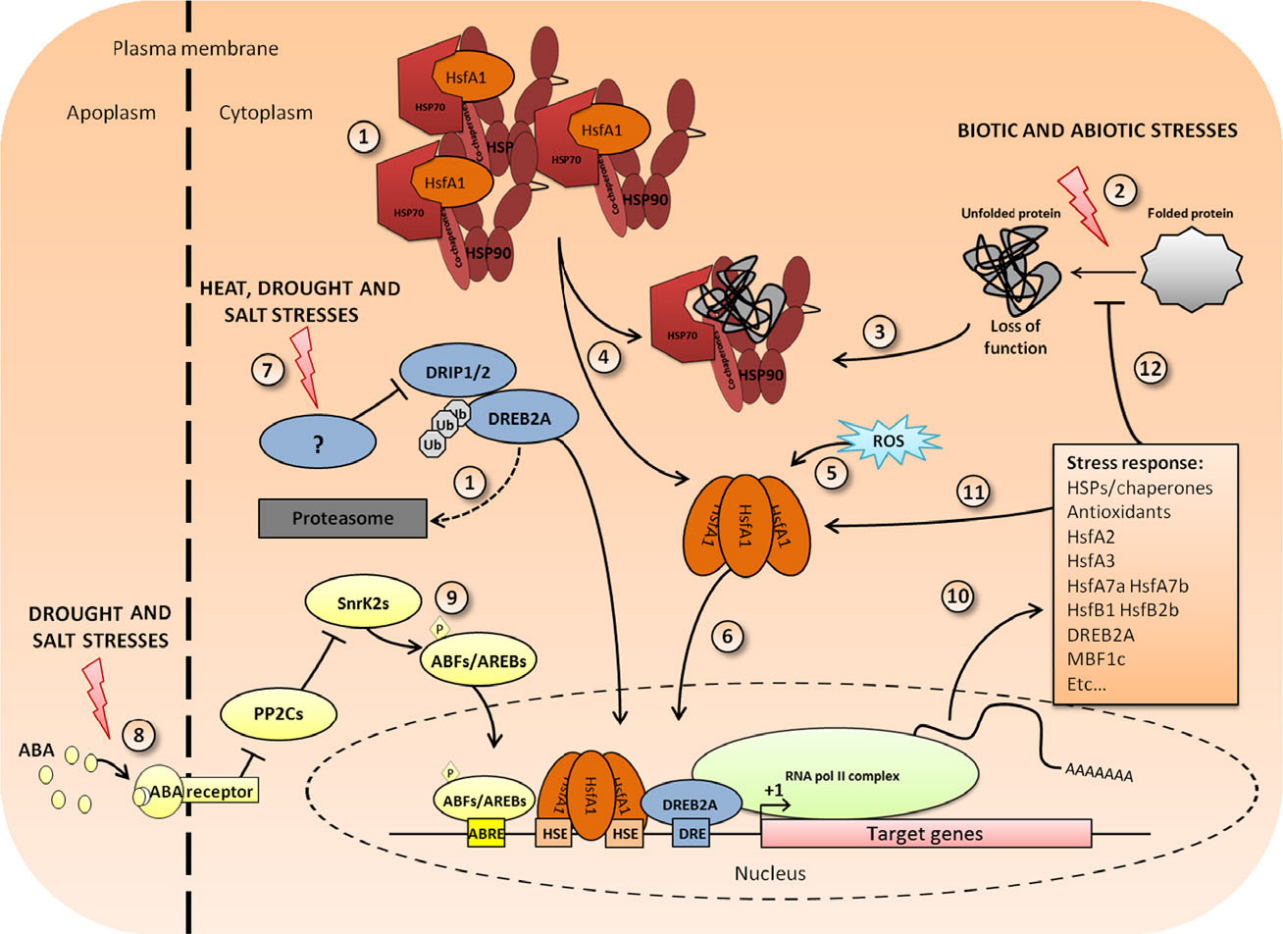
Schematic representation of the HSP/HSF pathway. (1) In nonstress conditions, class A1 HSFs are sequestered by HSP90/70 and their cochaperones and DREB2A is degraded through the UPS thanks to the E3 ligase DRIP1/2 (Qin *et al*., 2008). Upon stress application (2), the high number of misfolded proteins triggers the recruitment of HSP90/70 to its client and frees the HSFA1s following the chaperone titration model (4). In a high ROS context (5), the HSFA1s can form oligomers and are translocated in the nucleus (6) to bind HSE on DNA and induce target genes’ transcription. Trimers are represented here, in reference to mammalian HSF1 trimerization, even though the degree of oligomerization has not been established in plants except for AtHSFA1a trimerization. Other signalling pathways may interfere with the HSF/HSP pathway. Specific heat, drought and salinity stresses will lead to DRIP1/2 inhibition and DREB2A accumulation (7). Drought and salt stresses will induce ABA accumulation and binding to its receptor PYR/PYL/RCAR, leading to inactivation of PP2Cs (8). SnrK2s can then activate their target by phosphorylation (9). ABF/AREBs and DREB2A can then enter the nucleus, cooperatively or separately bind their target DNA motif, respectively, ABRE and DRE and HSE, to activate target genes’ expression (10). Induced proteins comprise stress‐specific ‘transcriptional relay’ TFs that feedback positively on HSF and HSP transcription (11) or proteins that participate in homeostasis re‐establishment (12). After the stress, the HSF/HSP content of the cell is different from the start. The quantity and the nature of the HSFs/HSP define the acclimated state.

The basic principles of the transcriptional control of HSPs are represented schematically in Figure 1. The main inducers of chaperones are heat‐shock factors (HSF), grouped into three classes A, B and C (for review, see Guo *et al*. (2016); Nover *et al*. (2001); Scharf *et al*. (2012)). HSFs are present in all eukaryotes, but plants show a large number of HSFs (38 in soya bean, 25 in rice, 21 in Arabidopsis) compared with a single HSF1 in *Saccharomyces cerevisiae* or with seven members in humans (Fujimoto and Nakai, 2010). The diversity of the HSF family in plants renders their study difficult. However, sequence and expression pattern comparisons showed both distinct and overlapping functions in stress resistance and development (von Koskull‐Döring *et al*., 2007).

There has been very few *in planta* study on B and C class HSFs. Class B and C HSFs lack the activator motif AHA (aromatic hydrophobic acidic) that is necessary for the transcriptional activity of class A HSFs and are therefore considered as inhibitory HSFs. Nevertheless, HSF B class members have been demonstrated to be absolutely necessary for the proper recovery from heat stress. In *Arabidopsis*, HSFB1 and HSFB2b have been shown to repress the induction of HSPs during stress recovery (Ikeda *et al*., 2011).

One unusual feature of HSF/HSP research is that many discoveries have been made on nonmodel species. It was first in tomato that the ‘master regulator’ *SlHSFA1* was identified. Transgenic plants overexpressing SlHSFA1 were found to be responding better to heat stress, whereas cosuppression (CS) lines were oversensitive to heat stress (Mishra *et al*., 2002).

In *Arabidopsis*, the transcription factors HSFA1abd and e are constitutively expressed and are responsible for triggering the HSR (heat stress response) (Yoshida *et al*., 2011) HSF1abde are responsible for basal thermotolerance and also initiate the acquisition of thermotolerance. These transcription factors bind to HSEs (heat stress elements) to activate transcription of *HSP*s as well as ‘transcriptional relay’ *HSF*s, mainly *HSFA2*,* HSFA3* and *HSFA7a*, that will, together with HSFA1 or separately, maintain a strong HSR during long‐term, repeated stresses. In this regard, HSFA2, HSFA3 and HSFA7a are considered as the most potent activators of *HSP* transcription during recovery. They are responsible for the heat‐acclimation phenotype (Charng *et al*., 2006; Nishizawa *et al*., 2006; Schramm *et al*., 2008). It is worth noting that *HSFA2* is the most highly heat‐induced HSF and *hsfa2* KO mutants are the only simple mutants which are completely unable to acquire thermotolerance. This is partly explained by the fact that HSFA2 is able to induce its own expression. However, the mechanism is not specific to *HSFA2* as other HSFs can also exert positive feedback on other HSFs. *HSFA3*,* HSFA7a* and *HSFA7b* are induced by HSFA2 and/or HSFA1s after heat stress (Liu and Charng, 2013). In turn, *HSFA1e* and *HSFA7b* and *HSFB2b* are induced by *HSFA3* overexpression (Yoshida *et al*., 2008). Similarly, HSFA6a overexpression induces *HSFA6b*,* HSFA5* and *HSFA2* (Hwang *et al*., 2014). Intriguingly, *HSFA3* overexpression in control conditions induces *HSFA1e* but not *HSFA2* expression*,* which is induced by HSFA1e after heat and/or high light stress. These complex interconnections and feedback loops demonstrate that multiple input signals can activate overlapping but different HSF/HSP responses.

This adapted HSF activity may also be a consequence of post‐translational modifications (PTMs). HSFA4a is a target of MPK3 and MPK6 (mitogen‐activated protein kinase). It was reported that phosphorylation by MPK3/6 increases the activity of HSFA4a (Pérez‐Salamó *et al*., 2014). It was further shown that HSFA2 phosphorylation by MPK6 is required for its nuclear localization, but the molecular mechanism determining the subcellular localization of HSFA2 has not been fully deciphered (Evrard *et al*., 2013). HSFA2 was also found to be sumoylated after heat stress (Cohen‐Peer *et al*., 2010) and an increased sumoylation was correlated with a decrease in HSFA2 activity and diminished *HSP* induction. SUMO1‐overexpressing plants showed an *hsfa2* KO phenotype with respect to heat stress tolerance. Most importantly, it is thought that homo/hetero‐oligomerization is very important for the modulation of HSP induction. In tomato, SlHSFB1 can positively or negatively regulate the transcription of *HSPs* by forming heterodimers with SlHSFA1. SlHSFA1‐SlHSFB1 can stimulate HSP production, whereas SlHSFB1 alone represses the transcription of *HSPs* (Hahn *et al*., 2011). SlHSFA1 activity is also enhanced by SlHSFA2 binding to such an extent that the SlHSFA1‐SlHSFA2 complex is called a ‘superactivator complex’ (Chan‐Schaminet *et al*., 2009). In *Arabidopsis*, despite of being an A class HSF, HSFA5 specifically binds to and inhibits AtHSFA4a. In mammals, HSF1 monomers are inactive and HSF1 is known to bind DNA as a homotrimer (for the detailed structure bound to DNA, see Neudegger *et al*. (2016)). Both in mammals and plants, ROS (reactive oxygen species) directly impact HSF oligomerization. ROS are acting as second messengers in a great variety of stresses and H_2_O_2_ induces binding of high molecular weight protein complexes on HSEs (Volkov *et al*., 2006) and trimerization of HSFA1a was shown to occur upon treatment with H_2_O_2_, heat or pH variation (Liu *et al*., 2013). ROS action on transcription factors often depends on the oxidation of one or several Cys residues, but a number of other amino acids could also be potential targets of H_2_O_2_ (reviewed in Driedonks *et al*. (2015)).

The amount of free HSPs is the sensor of the cell capacity to maintain a stable proteome and feeds back on its own production. Indeed, in unstressed tissues, the commonly accepted ‘chaperone titration model’ specifies that HSFs are sequestered by HSP70/90 and maybe other chaperones (Guo *et al*., 2001; Volkov *et al*., 2006). Strong evidences obtained in tomato suggest that an increase in SlHSP70/90 clients leads to liberating HSFs, allowing high HSP and HSF production. On the contrary, excess SlHSP70/90 will in turn favour SlHSFA1 inactivation and SlHSFB repression activity (Hahn *et al*., 2011). However, the sequestration of TFs by HSP70/90 may not always be a simple dose‐dependent effect but also a finely tuned process. In *Arabidopsis*, two FK506‐binding proteins (FKBPs), named ROF1 and ROF2, have been shown to regulate HSFA2 activity. ROF1 and 2 possess TPR (tetratricopeptide repeat) domains involved in HSP90 binding and domains involved in peptidyl‐prolyl cis‐trans isomerization of polypeptide bonds. It was demonstrated that ROF1 and 2 participate in the formation of an HSP90.1‐HSFA2 complex (Meiri *et al*., 2010). It was also observed that the HSFA2‐ROF1‐HSP90.1 complex keeps HSFA2 in a transcriptionally active state, whereas ROF2 binding inhibits HSFA2 activity. Interestingly, ROF2 is a target of HSFA2 produced 6 h after stress onset.

### HSF functional diversification

The large number of HSFs and the complex modulation of their activities by hetero‐oligomerization render the attribution of specific functions very difficult. In *Arabidopsis*, study of the different triple mutants *Hsfa1a, b, d*;* Hsfa1b, d, e*;* Hsfa1a, b, e* and *Hsfa1a, d, e* and also the quadruple *Hsfa1a, b, d, e* mutant revealed different specificity for stress resistance. The triple *Hsfa1a, b, d* and quadruple *Hsfa1a, b, d, e* mutants are unable to adapt to even moderately high temperatures. The *Hsfa1 b, d, e* quadruple mutant was hypersensitive to salt stress. All HSFA1s are involved in osmotic stress tolerance, with a preference for HSFA1d and HSFA1e. The presence of HSFA1b and d was sufficient for normal oxidative stress tolerance. The quadruple *Hsfa1a, b, d, e* mutant also showed a defect in seed development, exhibiting more than 20% abortion (Liu and Charng, 2013).

All of these defects were partially or completely rescued by overexpressing *HSFA2*. This is consistent with the finding that *HSFA2* is a target of HSFA1d and e. It has been thoroughly proven that overexpression of *HSFA2* is sufficient to enhance resistance to heat, anoxia, salt, osmotic stresses and a combination of heat, high light and oxidative stresses (Nishizawa *et al*., 2006; Ogawa *et al*., 2007). *HSFA2* expression can be induced by HSFA1d and e, but the double KO mutant does not display full inhibition of *HSFA2* expression during combined heat and high light stresses, suggesting that another factor than HSFA1d and e exists for regulating *HSFA2* expression (Nishizawa‐Yokoi *et al*., 2011). *HSFA2* is also involved in developmental processes. *HSFA2* overexpression increases callus proliferation (Ogawa *et al*., 2007). In tomato, SlHSFA2 is up‐regulated in anthers during pollen formation (Fragkostefanakis *et al*., 2016) and is important to mitigate pollen sensitivity to heat stress.

Apart from HSFA1s, several HSFs play a role in drought and salinity stress signalling. They can be induced by ABA‐dependent as well as ABA‐independent signalling pathways. The latter is represented by DREB (dehydration response element binding) TFs. *HSFA3* is a target of DREB2A (Yoshida *et al*., 2008), which is induced by multiple stresses such as drought, salt, heat and cold and to a lesser extent by oxidative stress, UV‐B light and wounding (Winter *et al*., 2007). DREB2A was first shown to be involved in drought and salt stress responses (Sakuma, 2006a). Consequently, DREB2A‐dependent *HSFA3* induction may not only be important for *HSP* production during thermotolerance but may also lead to salt and drought stress resistance. However, resistance to the above‐mentioned stresses was not investigated in HSFA3 overexpressing plants.

ABA‐dependent signalling relies on SnRK2's (sucrose nonfermenting related protein kinases 2) constitutive inhibition by PP2Cs (protein phosphatase 2 Cs; for review see (Singh and Laxmi, 2015)). PP2Cs are recruited to the ABA‐bound receptors PYR/PYL/RCARs (pyrabactin resistance/pyrabactin resistance 1‐like/regulatory component of ABA receptors), thereby releasing the inhibition of subclass III SnRK2s. The latter are controlling many ABA‐responsive transcription factors by phosphorylation, most importantly ABF1, ABF3 (ABA response factors 1 and 3), AREB1 and AREB2 (ABA‐responsive element‐binding proteins 1 and 2). Consequently, *snrk2d/e/i* triple mutant seeds are highly sensitive to humidity and are not able to induce seed‐specific expression of *HSFA7b* and *HSFA9* (Nakashima *et al*., 2009) in *Arabidopsis*. HSFA7b is present only after heat treatment and in drying seeds. The *HSFA7b* KO did not show defects in thermotolerance. Several HSFs are transiently induced during seed imbibition, namely *HSFA2*,* HSFA9*,* HSFA7b*,* HSFA7a*,* HSFB2a* and *HSFB2b* (Chiu *et al*., 2012). Most importantly, heat stress sustained the activation of these HSFs, leading to the accumulation of 49 HSP transcripts and is partially mediated by ABA. *HSFA9* is even more specific to seed protection processes as it is not inducible by stress. Its transcription is controlled by the seed‐specific ABA‐responsive transcription factor ABI3 (ABA insensitive 3) (Kotak *et al*., 2007). Interestingly, HSFA9 can interact with a component of auxin signalling, HaIAA27 (indole acetic acid) in sunflower (*Helianthus annuus*) (Carranco *et al*., 2010). Together with the seed abortion phenotype of the quadruple *Hsfa1a,b,d,e* mutant, these data highlight the importance of HSFs during seed development.

The promoter of *AtHSFA6a* contains two AREs (ABA‐responsive elements) and is bound *in vitro* by three ABA‐responsive TFs: AREB1, ABF3 and AREB3 (Hwang *et al*., 2014). Overexpressing plants exhibited resistance to salt and drought stresses. The fact that *HSFA6a* was not found to be a target of SnRK2‐dependent signalling may be due to a seed‐specific modulation of ABA signalling (Nakashima *et al*., 2009).

HSFB2b is a direct target of class III SnRK2s (i.e. ABA‐triggered SnRK2s), but it is not known to which extent this phosphorylation event is biologically relevant (Wang *et al*., 2013).

Similarly, *HSFA4a* was found to be induced transcriptionally during heat, salt, osmotic and cold stresses as well as during biotic stresses and *HSFA4a* overexpression leads to salt, oxidative and anoxia stress tolerance in Arabidopsis (Pérez‐Salamó *et al*., 2014).

Factors controlling the production of HSPs during biotic stresses are not well established. *AtHSFA2* and *AtHSFA7a* were shown to be involved in the CPR (cytoplasmic protein response), which is induced during the formation of large protein aggregates in the cytoplasm (Sugio *et al*., 2009). CPR is a feature of biotic stresses, as overexpression of viral proteins tends to overload the cellular machinery. In the same study, the induction of both HSFs was observed during *TuMV* (*Turnip mosaic virus*) or *TCV* (*Turnip crinkle virus*) infection, suggesting that HSFs are involved in virus resistance (Sugio *et al*., 2009). On the contrary, AtHSFB1 and AtHSFB2b negatively regulate the expression of the plant defensin genes PDF1.2a and b (Kumar *et al*., 2009). As a result, the simple mutant *Hsfb2b* and the double mutant *Hsfb1,b2* exhibited resistance to the necrotrophic fungus *Alternaria brassicicola*. Both B class HSFs are targets of HSFA1s and A2 (Liu and Charng, 2013). Intriguingly, overexpression of *AtHSFA1b* induces expression of *HSFB2b* but still protects plants from biotic, as well as drought and salt stresses (Bechtold *et al*., 2013).

HSFs were also shown massively recruited during oxidative stress. Oxidized lipids play an important role in the adaptation to oxidative stress. *HSFA1a*,* HSFA1e*,* HSFA2*,* HSFA4c*,* HSFA7a*,* HSFA8*,* HSFB1*,* HSFB3 HSFB4*,* HSFC1* and many *HSPs* are induced by OPDA (oxo phyto dienoic acid) treatment (Taki *et al*., 2005). Another oxidized lipid derivative was recently found to specifically induce the expression of genes of the HSP/chaperone network (Mata‐Pérez *et al*., 2016), including *HSFA2* and *HSFA7b*. Gene ontology (GO) annotation revealed that 21.25% of the genes up‐regulated by NO_2_‐Ln treatment were termed as ‘chaperones’, 21.25% as ‘response to heat’ and 6.88% as ‘heat acclimation’, suggesting that NO_2_‐Ln may play an important role in heat stress. The basis of oxylipin‐mediated induction of the HSP/chaperone network is still unknown.

### HSP/chaperones involvement in heat stress‐independent signalling

#### Biotic stress signalling

Several lines of evidence indicate that the HSP/HSF pathway is involved in biotic interactions (Park and Seo, 2015). HSP90 is strongly involved in resistance proteins (R proteins) stabilization and is necessary for proper defence signal transduction (Shirasu, 2009). Most human HSP90 clients are signalling components (Taipale *et al*., 2012) and it seems that similar principles apply to the plant kingdom (Iki *et al*., 2010; Ishiguro *et al*., 2002). HSP90 forms a complex with its co‐chaperones SGT1 and RAR1 (salicylic acid glucosyltransferase 1 and required for mla12 resistance 1). This complex is regulating important R proteins like RPM1 (resistance to *Pseudomonas maculicola* 1), RPS2, RPS4 (resistance to *Pseudomonas syringae* 2 and 4), RPP4 (recognition of *Peronospora parasitica* 4 (Bao *et al*. (2014) Hubert *et al*. (2003); Takahashi *et al*. (2003); Zhang *et al*. (2004)) and Rx (resistance to *Potato virus x*; Lu *et al*., 2003). Each HSP90 isoform has its own specificity. For example, *Hsp90.2* but not *Hsp90.3* mutants are especially sensitive to *Pseudomonas syringae pv tomato* (*Pst*) carrying the avirulence factor RPM1 (avrRPM1).

A role of HSP70 in pathogenesis is well illustrated by the study of Jelenska *et al*. (2010) on *Pst* infection. A *Pst* effector protein, HopI1, was reported to exert its virulence functions specifically on HSP70‐1 (Jelenska *et al*., 2010). Moreover, *Arabidopsis* plants with reduced levels of HSP70‐1 allowed enhanced growth of type III secretion‐deficient *Pst*. Altogether, these data established a specific role of HSP70‐1 in basal defence. On the contrary, heat‐shock cognate 70‐1 (HSC70‐1), a cytosolic and nuclear chaperone, was found to down‐regulate R protein‐mediated resistance to pathogens (Noël *et al*., 2007). The effect was attributed to the modulation of HSP90 functions in immunity, as HSP70 and HSP90 often cooperate in large multichaperone complexes (Li *et al*., 2012).

An HSP70‐specific resistance pathway exists. BiP (immunoglobulin‐binding protein) is an HSP70 isoform specifically located in the endoplasmic reticulum (ER) lumen. BiP has been linked with the UPR, which, like its cytoplasmic equivalent, is triggered under biotic stress conditions. The function of BiP in the UPR is reminiscent of HSP90/70‐dependent regulation of SlHSFA1/A2 in tomato. The accumulation of BiP clients induces the dissociation of BiP from the major UPR transducers IRE1 (inositol requiring enzyme 1), PERK [double‐stranded RNA‐activated protein kinase (PKR)‐like ER kinase] and ATF6 (activating transcription factor 6) (Bertolotti *et al*., 2000). However, it was found in yeast that IRE1 could induce normal UPR in the absence of BiP. BiP is now considered as a stabilizer of the UPR transducers, safeguarding the cell against unspecific UPR signalling (reviewed in Walter and Ron (2012)). Finally, *HSP101* transient overexpression in tobacco cells protects from *C. michiganensis‐*induced cell death (Shafikova *et al*., 2013), but not much is known on the involvement of other chaperones in biotic stress responses.

#### Drought stress signalling

Information about the involvement of HSP/chaperones in drought stress signalling is scarce. It was found that both the overexpression of *HSC70* and the use of a dominant negative (DN) form of *HSP90* disrupted ABA‐mediated stomata closure, thereby negatively affecting water loss in stress conditions. The impact of ABA treatment on *HSC70, HSP90*,* SGT1* and *RAR1* was investigated by Q‐PCR. A decrease in *SGT1a* mRNA and an increase in *HSC70‐4* mRNA was observed. *HSC70‐1* and *HSC70‐4* must therefore share the same function regarding the regulation of ABA signalling but under physiological conditions, only *HSC70‐4* is involved in mitigating ABA signals. Surprisingly, the opposite effect was observed for ABA‐mediated inhibition of germination. *HSP90* DN‐ and *HSC70*‐overexpressing seeds were strongly hypersensitive to ABA in this assay. The targets of HSP90 and HSC70 are not known yet but must be downstream of SnRK2 as it was fully activated after ABA treatment, despite the use of an HSP90 inhibitor (Clement *et al*., 2011).

#### Hormone signalling and development

HSP90 and its cochaperones also regulates diverse signal transduction proteins. MAPKs are involved in many biological processes, from stress responses to cell proliferation and development (reviewed in Colcombet and Hirt (2008)). Recently in tobacco, the SGT1‐HSP90 complex was found to mediate the hypersensitive response (HR) induced by MEK2^DD^ (a constitutively active form of MEK2, mitogen‐activated protein kinase kinase2 (Ichimura *et al*., 2016)). MEK2 regulates HR‐mediated pathogen resistance in tobacco and tomato (Oh and Martin, 2011). Interestingly, silencing of *HSP90* induced a drastic decrease in *MEK2* ^*DD*^ transcripts, while *SGT1* silencing destabilized MEK2^DD^, but not wild‐type MEK2 protein levels. Several evidences point to an involvement of the same complex in the regulation of MPK4. MEKK1 is necessary for the activation of MPK4 and functions in an antagonistic manner to MPK3 and MPK6. The *mekk1* mutant phenotype (dwarfism and H_2_O_2_ accumulation) was partially reverted at moderately high temperatures and further complemented by the *rar1* mutation at 26 °C (Ichimura *et al*., 2006). However, it is unclear where the HSP90‐RAR1 module acts in the signalling pathway. Moderately high temperatures were shown to inhibit overactivation of MPK3 and MPK6 in the *mekk1* mutant background. Chaperones may be needed to stabilize upstream positive regulators of MPK3/6 like MEK2^DD^.

*SHD* (*Shepherd*) is coding for an HSP90‐like protein residing in the ER. *shd* mutants exhibit defects similar to those induced in *clv* (*Clavata*) mutants. Like *clv* mutants, *shd* shows a disorganized shoot apical meristem (SAM), floral meristem (FM) and root apical meristem (RAM). Genetic analyses of *shd clv* and *shd wus* double mutants suggest that SHD is necessary for CLV signal transduction (Ishiguro *et al*., 2002).

The HSP90‐SGT1 complex is also involved in jasmonic acid (JA), auxin and gibberellic acid (GA) signalling. It was determined that SGT1a and b were necessary for the stable expression of COI1 (coronatine insensitive 1) and TIR1 (transport inhibitor response 1) proteins. An effect on GA signalling was indirectly determined by observing sustained overactivation of a GA down‐regulated gene (*CYP71A12*) after GA and Flg22 treatment (Zhang *et al*., 2015). Brassinosteroid (BR) signalling is also partially dependent on HSP90 as BES1 [brassinosteroid insensitive1 (BRI1) EMS suppressor1] is an HSP90 client (Lachowiec *et al*., 2013). The use of the well‐known HSP90 inhibitor geldanamycin showed that HSP90 is required for proper BR signalling, meaning that there could be more than one HSP90 client in the BR pathway. Unfortunately, no systematic analysis of HSP90 clients has been performed in plants.

Chaperones other than HSP90/70 can also modulate signal transduction events. The case of the *chaos* mutant is a good example of chaperone‐dependent canalization. The *CHAOS* locus is coding for a molecular chaperone named cpSRP43 (chloroplastic signal recognition particle of 43 kDa), involved in light harvesting complex proteins (LHCP) folding and chloroplast targeting. As LHCPs are intrinsic proteins, they are naturally prone to denaturation and absolutely require a chaperone for correct targeting and folding. It was shown that *cpSRP43* is down‐regulated during cold and high light stress acclimation (Klenell *et al*., 2005). The consequent decrease in LHCP levels reduced ROS production and sensitivity to oxidative stress. Moreover, *chaos* mutants were constitutively acclimated to cold and were performing better than WT under repeated stress conditions (Klenell *et al*., 2005).

Chaperones of the HSP100 family play a crucial role in development. HSP100 chaperones are essential components of the protein quality control (PQC) process. They act in concert with HSP70 chaperones to thread and degrade toxic protein aggregates (Mogk *et al*., 2015). Major metabolic pathways require these HSP100s to reactivate or degrade misfolded enzymes following environmental stresses (Pulido *et al*., 2016). They are also involved in protein targeting as they process the signal peptide of specific precursor proteins once they have reached their destination. For instance, ClpC1 was shown to be involved specifically in photosystem biogenesis. In the *clpc1* mutant, chlorophyll, PSI and PSII levels were down‐regulated and growth was consequently strongly impaired (Sjögren *et al*., 2004). HSP100 chaperone's role is not limited to proteins. HSP101 was shown to bind a nucleotide sequence in the 5′UTR (untranslated region) of some mRNAs. It was demonstrated that HSP101 was able to stabilize target mRNAs and enhance their translation (Ling *et al*., 2000; Wells *et al*., 1998; Wu *et al*., 2013).

### Engineering the HSFs for multistress resistance

The HSP/chaperone pathway is exerting pleiotropic regulation of gene expression on both the translational and post‐translational levels. In addition to protect a broad spectrum of proteins, chaperones regulate a great number of signal transduction components. Four major features define the HSP/chaperone pathway: it is *ubiquitous*, able to *memorize* stress, it can respond to a variety of stresses thanks to a great degree of *modularity* and shows complex *feedback loops*, both positive and negative. The HSP/chaperone pathway provides stability both in control and stress conditions. As a consequence, it should be considered as a key actor of canalization. The impact of HSF overexpression is varied. It was demonstrated that the overexpression of *AtHSFA1b*,* AtHSFA2*,* AtHSFA3*,* AtHsfA4a* and *AtHSFA6a* induces chaperone production and improves stress resistance in *Arabidopsis*. Nonetheless, it also modulated developmental programs (reviewed in Fragkostefanakis *et al*. (2014)).

*DREB2A* modulates *HSFA3* expression, and deletion of the regulatory domain of DREB2A leads to the production of a CA (constitutively active) form of the protein. The resulting plants constitutively express *HSFA3* and exhibit resistance to drought, salt and heat stresses. Nonetheless, transgenic plants displayed growth retardation (Sakuma *et al*., 2006b). *AtHSFA3* overexpression has been shown to elevate thermotolerance but also produced moderate to severe dwarfism (Yoshida *et al*., 2008). The impact of *AtHSFA3* overexpression on other stresses was not investigated. Tomato *SlHSFA3* overexpression in *Arabidopsis* gave a different phenotype. Plants were resistant to heat but more sensitive to salt stress. Flowering was also delayed, but they were not dwarf as 35S::*AtHSFA3* plants (Li *et al*., 2013). It would be interesting to compare SlHSFA3 and AtHSFA3 targets to identify genes responsible for the dwarf phenotype.

Constitutive induction of *HSFA4a* was detrimental to plants as *HSFA4a* overexpression showed 20%–30% growth reduction in control conditions. However, when exposed to salt, anoxia or oxidative stress, HSFA4a‐overexpressing plants performed better than control. Expression of *HSFA4a* was induced by numerous other stresses like UV‐B, cold, drought ozone or pathogens (Pérez‐Salamó *et al*., 2014). The impact of *HSFA4a* overexpression on resistance to those stresses was not investigated but could be very interesting.

*AtHSFA6a* and *AtHSFA6b* are not induced by heat stress treatment but are specifically induced by ABA, salt, drought and osmotic stress (Huang *et al*., 2014, 2016; Hwang *et al*., 2014). Consistently, the promoter of *AtHSFA6a* contains two ARE and was bound *in vitro* by three ABA‐responsive TFs: AREB1, ABF3 and AREB3. Similarly, *AtHSFA6b* promoter contains several ABREs bound *in vitro* by AREB1. Overexpressing plants exhibited resistance to salt and drought stress. A genetic screen aiming at discovering mutations inducing constitutive expression of *HSFA6a* and *AtHSFA6b* would be promising.

*HSFA1b* is particularly promising with respect to its multistress resistance potential. *HSFA1b* overexpression confers resistance to drought, salt and biotic stress and enhances seed yield (Bechtold *et al*., 2013). Analysis of an HSFB1b‐specific heat‐shock element in the promoter of *HSFA1b*‐differentially expressed genes (DEGs) allowed the identification of 55 direct targets among the DEGs. Among those 55 genes were several transcription factors related to multiple stresses (in particular *HSFA7A*,* HSFB2b*,* HSFB2a* and *MBF1c*) that should be responsible for the resistance traits (Bechtold *et al*., 2013).

The particular role of HSFA2 as a response amplifier and in stress acclimation makes it a strong candidate for resistance engineering. A relatively small overexpression of *AtHSFA2* did not negatively impact biomass but led to a weak resistance to heat stress, oxidative stress and a combination of heat, high light and oxidative stresses (Li *et al*., 2005; Nishizawa *et al*., 2006). High‐level overexpression of *HSFA2* was achieved with the El2Ω promoter, resulting in a 400‐fold increase in *HSFA2* expression. El2Ω::*AtHSFA2* plants displayed dwarfism as well as resistance to heat, osmotic and salt stresses (Ogawa *et al*., 2007). Nevertheless, this difficulty might be overcome using inducible instead of constitutive promoters. Interestingly, overexpression of *SlHSFA1* in tomato (which constitutively activates *SlHSFA2*) led to an increase in biomass as well as heat stress resistance (Mishra *et al*., 2002). This observation suggests that it should be possible to uncouple HSF‐mediated resistance and growth inhibition. A genetic screen aiming at reverting the dwarf phenotype of El2Ω::*AtHSFA2* would be as difficult as it would be interesting.

The production of transgenic plants with altered HSF/HSP expression has also been employed to produce stress resistance in crops. Overexpression of endogenous HSF has been performed in tomato (Mishra *et al*., 2002) and soybean (Zhu *et al*., 2006), successfully increasing plant tolerance to stress. However, the HSF families in crop species are also diverse and crop transformation is time‐consuming. Overexpression of one, randomly chosen, HSF could be inadequate to increase resistance to a specific set of stresses. To overcome these difficulties, several HSFs from wheat or rice were first characterized in transgenic *Arabidopsis*. For instance, a role of *OsHSF7*,* OsHSFA2a* and *TaHSFA2d* in stress responses has been confirmed in *Arabidopsis* (Chauhan *et al*., 2013; Liu *et al*., 2009; Yokotani *et al*., 2008). These genes could now be used for stable *in planta* overexpression in crop plants.

### Future perspectives of technology transfer to crops

Generally speaking, it seems that the most promising targets in terms of enhancing stress resistance are the most upstream components of a given signalling pathway, as exemplified by *AtHSFA1b* and *SlHSFA1*. It may be that activating a whole branch of a signalling pathway allows a better tuning of the response than activating just one downstream element. Another promising way to increase resistance would require understanding the principles of growth arrest in stress‐resistant plants. It may be possible to uncouple the functions of growth inhibition from those of stress protection in a given signalling pathway. A genetic screen aiming at finding a reversion of the dwarfism of *HSFA2* or *HSFA3* overexpressors could lead to mutants that disconnect resilience and growth.

Still, will these results be applicable to crop resistance engineering? Several lines of evidence point towards a broad conservation of the HSP/chaperone network as a multiple stress protection mechanism among all land plants. Indeed, plant land adaptation was shown to be strongly dependent on HSPs as exemplified by the diversification of *HSP70*. The green algae *Chlamydomonas reinhardtii* possesses only six *HSP70* isoforms, whereas *Physcomitrella patens*,* Oryza sativa* and *Populus trichocarpa* have, respectively, 21, 24 and 20 *HSP70* isoforms (Tang *et al*., 2016). Gene expression analyses revealed moss *HSP70* isoforms were responsive to ABA, drought and salt stresses, in addition to heat stress (Tang *et al*., 2016). Gene overexpression and knockout studies showed *HSP70s* from tobacco, soya bean and citrus play a protective role during dehydration stress (Yu *et al*., 2015). In rice, *OsAHL1* was shown to directly induce *HSP101* and *HSP90* expression leading to drought resistance (Zhou *et al*., 2016). *OsHSP90‐2* and *OsHSP90‐4* were also found up‐regulated after drought, cold, heat and salt stresses (Zhang *et al*., 2016). When introduced in *E. coli*,* OsHSP90‐2* was sufficient to induce resistance to heat, high salinity and drought (Zhang *et al*., 2016). A similar modulation of *HSFs* and *HSPs* was found in tomato in response to heat, drought and salinity (Fragkostefanakis *et al*., 2015). In wheat, overexpression of *Triticum aestivum HSFA6f* was demonstrated to direct the expression of several *HSPs*, leading to thermotolerance (Xue *et al*., 2015). Most strikingly, the expression of a maize *HSF*,* ZmHSF06* (*Zea mays HSF06*), is sufficient to confer heat and drought stress resistance to *Arabidopsis* (Li *et al*., 2015). The conservation of HSF signalling from monocots to dicots definitely provides evidence for a strong conservation of HSP‐based multiple stress responses in crops.

From the above‐mentioned facts, it is clear that many good targets to enhance multistress resistance are defined. Nonetheless, the technology is not readily transposable to crop species. Up to now, most attempts to enhance resistance have used transgenic 35S promoter‐driven overexpression of class A HSFs, sometimes from stress‐resistant species. However, two major obstacles restrict the use of this technique. Firstly, GMOs are ‘associated with unnaturalness and trigger disgust’ (Blancke *et al*., 2015) in the general population and this feeling is now translated into the official European regulation. Even though the ban on GMOs is now limited to Europe, it is a rising concern worldwide and must be considered carefully. Secondly and most importantly, 35S‐driven overexpression is unreliable. It does not produce a normally distributed high‐level expression of genes and may be subjected to gene silencing (Butaye *et al*., 2004). Performing a genetic screen to identify *Arabidopsis* mutants with enhanced *HSF* expression (using a reporter gene to monitor HSFs’ activity) would be a more adequate approach. The TILLING (targeted induced local lesion in genomes) technique can then be used to apply knowledge gained from *Arabidopsis* directly into many cultivated species. Numerous mutant populations of crop species already exist and can be screened for a specific variant. Mutant *loci* can be identified in a matter of weeks thanks to the massive expansion of NGS (next generation sequencing) techniques (Kurowska *et al*., 2011). The recently discovered CRISPR‐CAS9 (clustered regularly interspaced short palindromic repeat‐CRISPR associated 9) system would also allow a rapid technology transfer in crops. The advantage of CRISPR‐CAS9 compared with TILLING would be that the latter requires a significant number of crosses to introgress one mutation in a competitive cultivar. Also, mutagenic agents used in TILLING introduce many unwanted mutations in the genetic background that need to be ‘cleaned’. In this regard, genome editing by CRISPR‐CAS9 would be cleaner and faster (Bortesi and Fischer, 2015). Nonetheless, CRISPR‐CAS9 still relies on transgenic DNA insertion and may be considered as a GMO and subjected to the same regulation, even though the end product does not contain any transgene. Regardless, mutations enhancing HSF expression or activity undoubtedly are valuable targets to engineer multistress‐resistant crops.

## Conflict of interest

The authors declare no conflict of interest, in accordance with the policy described in the Instructions for Author (http://onlinelibrary.wiley.com/journal/10.1111/%28ISSN%291467-7652/homepage/ForAuthors.html).

## References

[pbi12659-bib-0001] Al‐Whaibi, M.H. (2011) Plant heat‐shock proteins: a mini review. J. King Saud University – Sci. 23, 139–150.

[pbi12659-bib-0002] Bao, F. , Huang, X. , Zhu, C. , Zhang, X. , Li, X. and Yang, S. (2014) Arabidopsis HSP90 protein modulates RPP4‐mediated temperature‐dependent cell death and defense responses. New Phytol. 202, 1320–1334. 24611624 10.1111/nph.12760

[pbi12659-bib-0003] Bechtold, U. , Albihlal, W.S. , Lawson, T. , Fryer, M.J. , Sparrow, P.A.C. , Richard, F. , Persad, R. *et al*. (2013) Arabidopsis HEAT SHOCK TRANSCRIPTION FACTORA1b overexpression enhances water productivity, resistance to drought, and infection. J. Exp. Bot. 64, 3467–3481. 23828547 10.1093/jxb/ert185 PMC3733161

[pbi12659-bib-0004] Bertolotti, A. , Zhang, Y. , Hendershot, L.M. , Harding, H.P. and Ron, D. (2000) Dynamic interaction of BiP and ER stress transducers in the unfolded‐protein response. Nat. Cell Biol. 2, 326–332. 10854322 10.1038/35014014

[pbi12659-bib-0005] Blancke, S. , Van Breusegem, F. , De Jaeger, G. , Braeckman, J. and Van Montagu, M. (2015) Fatal attraction: the intuitive appeal of GMO opposition. Trends Plant Sci. 7, 414–418. 10.1016/j.tplants.2015.03.011 25868652

[pbi12659-bib-0006] Bortesi, L. and Fischer, R. (2015) The CRISPR/Cas9 system for plant genome editing and beyond. Biotechnol. Adv. 33, 41–52. 25536441 10.1016/j.biotechadv.2014.12.006

[pbi12659-bib-0007] Butaye, K.M.J. , Goderis, I.J.W.M. , Wouters, P.F.J. , Pues, J.M.T.G. , Delauré, S.L. , Broekaert, W.F. , Depicker, A. *et al*. (2004) Stable high‐level transgene expression in Arabidopsis thaliana using gene silencing mutants and matrix attachment regions. Plant J. 39, 440–449. 15255872 10.1111/j.1365-313X.2004.02144.x

[pbi12659-bib-0008] Carey, C.C. , Gorman, K.F. and Rutherford, S. (2006) Modularity and intrinsic evolvability of Hsp90‐buffered change. PLoS ONE, 1, 1–6. 10.1371/journal.pone.0000076 PMC1762356 17183708

[pbi12659-bib-0009] Carranco, R. , Espinosa, J.M. , Prieto‐Dapena, P. , Almoguera, C. and Jordano, J. (2010) Repression by an auxin/indole acetic acid protein connects auxin signaling with heat shock factor‐mediated seed longevity. Proc. Natl Acad. Sci. USA, 107, 21908–21913. 21115822 10.1073/pnas.1014856107 PMC3003009

[pbi12659-bib-0010] Chan‐Schaminet, K.Y. , Baniwal, S.K. , Bublak, D. , Nover, L. and Scharf, K.D. (2009) Specific interaction between tomato HsfA1 and HsfA2 creates hetero‐oligomeric superactivator complexes for synergistic activation of heat stress gene expression. J. Biol. Chem. 284, 20848–20857. 19491106 10.1074/jbc.M109.007336 PMC2742850

[pbi12659-bib-0011] Charng, Y.Y. , Liu, H.C. , Liu, N.Y. , Chi, W.T. , Wang, C.N. , Chang, S.H. and Wang, T.T. (2006) A heat‐inducible transcription factor, HsfA2, is required for extension of acquired thermotolerance in arabidopsis. Plant Physiol. 143, 251–262. 17085506 10.1104/pp.106.091322 PMC1761974

[pbi12659-bib-0012] Chauhan, H. , Khurana, N. , Agarwal, P. , Khurana, J.P. and Khurana, P. (2013) A seed preferential heat shock transcription factor from wheat provides abiotic stress tolerance and yield enhancement in transgenic Arabidopsis under heat stress environment. PLoS ONE, 8, e79577. 24265778 10.1371/journal.pone.0079577 PMC3827158

[pbi12659-bib-0013] Chiu, R.S. , Nahal, H. , Provart, N.J. and Gazzarrini, S. (2012) The role of the Arabidopsis FUSCA3 transcription factor during inhibition of seed germination at high temperature. BMC Plant Biol. 12, 15. 22279962 10.1186/1471-2229-12-15 PMC3296646

[pbi12659-bib-0014] Clement, M. , Leonhardt, N. , Droillard, M.J. , Reiter, I. , Montillet, J.L. , Genty, B. , Lauriere, C. *et al*. (2011) The cytosolic/nuclear HSC70 and HSP90 molecular chaperones are important for stomatal closure and modulate abscisic acid‐dependent physiological responses in Arabidopsis. Plant Physiol. 156, 1481–1492. 21586649 10.1104/pp.111.174425 PMC3135925

[pbi12659-bib-0015] Cohen‐Peer, R. , Schuster, S. , Meiri, D. , Breiman, A. and Avni, A. (2010) Sumoylation of Arabidopsis heat shock factor A2 (HsfA2) modifies its activity during acquired thermotholerance. Plant Mol. Biol. 74, 33–45. 20521085 10.1007/s11103-010-9652-1

[pbi12659-bib-0016] Colcombet, J. and Hirt, H. (2008) Arabidopsis MAPKs: a complex signalling network involved in multiple biological processes. Biochem. J. 413, 217–226. 18570633 10.1042/BJ20080625

[pbi12659-bib-0017] Driedonks, N. , Xu, J. , Peters, J.L. , Park, S. and Rieu, I. (2015) Multi‐level interactions between heat shock factors, heat shock proteins, and the redox system regulate acclimation to heat. Front. Plant Sci. 6, 999. 26635827 10.3389/fpls.2015.00999 PMC4647109

[pbi12659-bib-0018] Evrard, A. , Kumar, M. , Lecourieux, D. , Lucks, J. , von Koskull‐Döring, P. and Hirt, H. (2013) Regulation of the heat stress response in Arabidopsis by MPK6‐targeted phosphorylation of the heat stress factor HsfA2. PeerJ, 1, e59. 23638397 10.7717/peerj.59 PMC3628891

[pbi12659-bib-0019] Fragkostefanakis, S. , Röth, S. , Schleiff, E. and Scharf, K.D. (2014) Prospects of engineering thermotolerance in crops through modulation of heat stress transcription factor and heat shock protein networks. Plant, Cell Environ. 38, 1881–1895. 24995670 10.1111/pce.12396

[pbi12659-bib-0020] Fragkostefanakis, S. , Simm, S. , Paul, P. , Bublak, D. , Scharf, K.D. and Schleiff, E. (2015) Chaperone network composition in Solanum lycopersicum explored by transcriptome profiling and microarray meta‐analysis. Plant, Cell Environ. 38, 693–709. 25124075 10.1111/pce.12426

[pbi12659-bib-0021] Fragkostefanakis, S. , Mesihovic, A. , Simm, S. , Paupière, M.J. , Hu, Y. , Paul, P. , Mishra, S.K. *et al*. (2016) HsfA2 controls the activity of developmentally and stress‐regulated heat stress protection mechanisms in tomato male reproductive tissues. Plant Physiol. 170, 2461–2477. 26917685 10.1104/pp.15.01913 PMC4825147

[pbi12659-bib-0022] Fu, X. (2014) Chaperone function and mechanism of small heat‐shock proteins. Acta Biochim. Biophys. Sin. 46, 347–356. 24449783 10.1093/abbs/gmt152

[pbi12659-bib-0023] Fujimoto, M. and Nakai, A. (2010) The heat shock factor family and adaptation to proteotoxic stress. FEBS J. 277, 4112–4125. 20945528 10.1111/j.1742-4658.2010.07827.x

[pbi12659-bib-0024] Guo, Y. , Guettouche, T. , Fenna, M. , Boellmann, F. , Pratt, W.B. , Toft, D.O. , Smith, D.F. *et al*. (2001) Evidence for a mechanism of repression of heat shock factor 1 transcriptional activity by a multichaperone complex. J. Biol. Chem. 276, 45791–45799. 11583998 10.1074/jbc.M105931200

[pbi12659-bib-0025] Guo, M. , Liu, J.‐H. , Ma, X. , Luo, D.‐X. , Gong, Z.‐H. and Lu, M.‐H. (2016) The plant heat stress transcription factors (HSFs): structure, regulation, and function in response to abiotic stresses. Front. Plant Sci. 7, 114. 26904076 10.3389/fpls.2016.00114 PMC4746267

[pbi12659-bib-0026] Hahn, A. , Bublak, D. , Schleiff, E. and Scharf, K.‐D. (2011) Crosstalk between Hsp90 and Hsp70 chaperones and heat stress transcription factors in tomato. Plant Cell, 23, 741–755. 21307284 10.1105/tpc.110.076018 PMC3077788

[pbi12659-bib-0027] Huang, S. , Monaghan, J. , Zhong, X. , Lin, L. , Sun, T. , Dong, O.X. and Li, X. (2014) HSP90s are required for NLR immune receptor accumulation in Arabidopsis. Plant J. 79, 427–439. 24889324 10.1111/tpj.12573

[pbi12659-bib-0028] Huang, Y.‐C. , Niu, C.‐Y. , Yang, C.‐R. and Jinn, T.‐L. (2016) The heat‐stress factor HSFA6b connects ABA signaling and ABA‐mediated heat responses. Plant Physiol. 4, 00860. 10.1104/pp.16.00860 PMC5047099 27493213

[pbi12659-bib-0029] Hubert, D.A. , Tornero, P. , Belkhadir, Y. , Krishna, P. , Takahashi, A. , Shirasu, K. and Dangl, J.L. (2003) Cytosolic HSP90 associates with and modulates the Arabidopsis RPM1 disease resistance protein. EMBO J. 22, 5679–5689. 14592967 10.1093/emboj/cdg547 PMC275404

[pbi12659-bib-0030] Hwang, S.M. , Kim, D.W. , Woo, M.S. , Jeong, H.S. , Son, Y.S. , Akhter, S. , Choi, G.J. *et al*. (2014) Functional characterization of Arabidopsis HsfA6a as a heat‐shock transcription factor under high salinity and dehydration conditions. Plant, Cell Environ. 37, 1202–1222. 24313737 10.1111/pce.12228

[pbi12659-bib-0031] Ichimura, K. , Casais, C. , Peck, S.C. , Shinozaki, K. and Shirasu, K. (2006) MEKK1 is required for MPK4 activation and regulates tissue‐specific and temperature‐dependent cell death in Arabidopsis. J. Biol. Chem. 281, 36969–36976. 17023433 10.1074/jbc.M605319200

[pbi12659-bib-0032] Ichimura, K. , Shinzato, T. , Edaki, M. , Yoshioka, H. and Shirasu, K. (2016) SGT1 contributes to maintaining protein levels of MEK2DD to facilitate hypersensitive response‐like cell death in Nicotiana benthamiana. Physiol. Mol. Plant Pathol. 94, 47–52.

[pbi12659-bib-0033] Ikeda, M. , Mitsuda, N. and Ohme‐Takagi, M. (2011) Arabidopsis HsfB1 and HsfB2b act as repressors of the expression of heat‐inducible Hsfs but positively regulate the acquired thermotolerance. Plant Physiol. 157, 1243–1254. 21908690 10.1104/pp.111.179036 PMC3252156

[pbi12659-bib-0034] Iki, T. , Yoshikawa, M. , Nishikiori, M. , Jaudal, M.C. , Matsumoto‐Yokoyama, E. , Mitsuhara, I. , Meshi, T. *et al*. (2010) *In vitro* assembly of plant RNA‐induced silencing complexes facilitated by molecular chaperone HSP90. Mol. Cell, 39, 282–291. 20605502 10.1016/j.molcel.2010.05.014

[pbi12659-bib-0035] Ishiguro, S. , Watanabe, Y. , Ito, N. , Nonaka, H. , Takeda, N. , Sakai, T. , Kanaya, H. *et al*. (2002) SHEPHERD is the Arabidopsis GRP94 responsible for the formation of functional CLAVATA proteins. EMBO J. 21, 898–908. 11867518 10.1093/emboj/21.5.898 PMC125899

[pbi12659-bib-0036] Jelenska, J. , van Hal, J.A. and Greenberg, J.T. (2010) Pseudomonas syringae hijacks plant stress chaperone machinery for virulence. Proc. Natl Acad. Sci. USA, 107, 13177–13182. 20615948 10.1073/pnas.0910943107 PMC2919979

[pbi12659-bib-0037] Kadota, Y. and Shirasu, K. (2012) The HSP90 complex of plants. Biochimica et Biophysica Acta – Molecul. Cell Res. 3, 689–697. 10.1016/j.bbamcr.2011.09.016 22001401

[pbi12659-bib-0038] Klenell, M. , Morita, S. , Tiemblo‐Olmo, M. , Mühlenbock, P. , Karpinski, S. and Karpinska, B. (2005) Involvement of the chloroplast signal recognition particle cpSRP43 in acclimation to conditions promoting photooxidative stress in Arabidopsis. Plant Cell Physiol. 46, 118–129. 15659446 10.1093/pcp/pci010

[pbi12659-bib-0039] von Koskull‐Döring, P. , Scharf, K.‐D. and Nover, L. (2007) The diversity of plant heat stress transcription factors. Trends Plant Sci. 12, 452–457. 17826296 10.1016/j.tplants.2007.08.014

[pbi12659-bib-0040] Kotak, S. , Vierling, E. , Baumlein, H. and Koskull‐Doring, P.V. (2007) A novel transcriptional cascade regulating expression of heat stress proteins during seed development of Arabidopsis. Plant Cell, 19, 182–195. 17220197 10.1105/tpc.106.048165 PMC1820961

[pbi12659-bib-0041] Kriechbaumer, V. , von Löffelholz, O. and Abell, B.M. (2012) Chaperone receptors: guiding proteins to intracellular compartments. Protoplasma, 1, 21–30. 10.1007/s00709-011-0270-9 21461941

[pbi12659-bib-0042] Krukenberg, K.A. , Street, T.O. , Lavery, L.A. and Agard, D.A. (2011) Conformational dynamics of the molecular chaperone Hsp90. Q. Rev. Biophys. 44, 229–255. 21414251 10.1017/S0033583510000314 PMC5070531

[pbi12659-bib-0043] Kumar, M. , Busch, W. , Birke, H. , Kemmerling, B. , Nürnberger, T. and Schöffl, F. (2009) Heat shock factors HsfB1 and HsfB2b are involved in the regulation of Pdf1.2 expression and pathogen resistance in Arabidopsis. Mol. Plant, 2, 152–165. 19529832 10.1093/mp/ssn095 PMC2639743

[pbi12659-bib-0044] Kurowska, M. , Daszkowska‐Golec, A. , Gruszka, D. , Marzec, M. , Szurman, M. , Szarejko, I. and Maluszynski, M. (2011) TILLING – a shortcut in functional genomics. J. Appl. Genet. 52, 371–390. 21912935 10.1007/s13353-011-0061-1 PMC3189332

[pbi12659-bib-0045] Lachowiec, J. , Lemus, T. , Thomas, J.H. , Murphy, P.J.M. , Nemhauser, J.L. and Queitsch, C. (2013) The protein chaperone HSP90 can facilitate the divergence of gene duplicates. Genetics, 193, 1269–1277. 23410833 10.1534/genetics.112.148098 PMC3606102

[pbi12659-bib-0046] Lee, S. , Lee, D.W. , Lee, Y. , Mayer, U. , Stierhof, Y.‐D. , Lee, S. , Jürgens, G. *et al*. (2009) Heat shock protein cognate 70‐4 and an E3 ubiquitin ligase, CHIP, mediate plastid‐destined precursor degradation through the ubiquitin‐26S proteasome system in Arabidopsis. Plant Cell, 21, 3984–4001. 20028838 10.1105/tpc.109.071548 PMC2814507

[pbi12659-bib-0047] Li, C.G. , Chen, Q.J. , Gao, X.Q. , Qi, B.S. , Chen, N.Z. , Xu, S.M. , Chen, J. *et al*. (2005) AtHsfA2 modulates expression of stress responsive genes and enhances tolerance to heat and oxidative stress in Arabidopsis. Sci. China Series C‐Life Sci. 48, 540–550. 10.1360/062005-119 16483133

[pbi12659-bib-0048] Li, J. , Soroka, J. and Buchner, J. (2012) The Hsp90 chaperone machinery: conformational dynamics and regulation by co‐chaperones. Biochimica et Biophysica Acta – Mol. Cell Res. 3, 624–635. 10.1016/j.bbamcr.2011.09.003 21951723

[pbi12659-bib-0049] Li, Z. , Zhang, L. , Wang, A. , Xu, X. and Li, J. (2013) Ectopic overexpression of SlHsfA3, a heat stress transcription factor from tomato, confers increased thermotolerance and salt hypersensitivity in germination in transgenic arabidopsis. PLoS ONE, 8, e54880. 23349984 10.1371/journal.pone.0054880 PMC3551807

[pbi12659-bib-0050] Li, H.‐C. , Zhang, H.‐N. , Li, G.‐L. , Liu, Z.‐H. , Zhang, Y.‐M. , Zhang, H.‐M. and Guo, X.‐L. (2015) Expression of maize heat shock transcription factor gene *ZmHsf06* enhances the thermotolerance and drought‐stress tolerance of transgenic *Arabidopsis* . Funct. Plant Biol. 42, 1080–1091. 32480747 10.1071/FP15080

[pbi12659-bib-0051] Ling, J. , Wells, D.R. , Tanguay, R.L. , Dickey, L.F. , Thompson, W.F. and Gallie, D.R. (2000) Heat shock protein HSP101 binds to the fed‐1 internal light regulator y element and mediates its high translational activity. Plant Cell Online, 12, 1213–1228. 10.1105/tpc.12.7.1213 PMC149060 10899985

[pbi12659-bib-0052] Liu, H.‐C. and Charng, Y.‐Y. (2013) Common and distinct functions of Arabidopsis class A1 and A2 heat shock factors in diverse abiotic stress responses and development. Plant Physiol. 163, 276–290. 23832625 10.1104/pp.113.221168 PMC3762648

[pbi12659-bib-0053] Liu, J.‐G. , Qin, Q.‐L. , Zhang, Z. , Peng, R.‐H. , Xiong, A.‐S. , Chen, J.‐M. and Yao, Q.‐H. (2009) OsHSF7 gene in rice, Oryza sativa L., encodes a transcription factor that functions as a high temperature receptive and responsive factor. BMB Reports, 42, 16–21. 19192388 10.5483/bmbrep.2009.42.1.016

[pbi12659-bib-0054] Liu, Y. , Zhang, C. , Chen, J. , Guo, L. , Li, X. , Li, W. , Yu, Z. *et al*. (2013) Arabidopsis heat shock factor HsfA1a directly senses heat stress, pH changes, and hydrogen peroxide via the engagement of redox state. Plant Physiol. Biochem. 64, 92–98. 23399534 10.1016/j.plaphy.2012.12.013

[pbi12659-bib-0055] Lu, R. , Malcuit, I. , Moffett, P. , Ruiz, M.T. , Peart, J. , Wu, A.‐J. , Rathjen, J.P. *et al*. (2003) High throughput virus‐induced gene silencing implicates heat shock protein 90 in plant disease resistance. EMBO J. 22, 5690–5699. 14592968 10.1093/emboj/cdg546 PMC275403

[pbi12659-bib-0056] Maaroufi, H. and Tanguay, R.M. (2013) Analysis and phylogeny of small heat shock proteins from marine viruses and their cyanobacteria host. PLoS ONE, 8, e81207. 24265841 10.1371/journal.pone.0081207 PMC3827213

[pbi12659-bib-0057] Mata‐Pérez, C. , Sánchez‐Calvo, B. , Padilla, M.N. , Begara‐Morales, J.C. , Luque, F. , Melguizo, M. , Jiménez‐Ruiz, J. *et al*. (2016) Nitro‐fatty acids in plant signaling: nitro‐linolenic acid induces the molecular chaperone network in arabidopsis. Plant Physiol. 170, 686–701. 26628746 10.1104/pp.15.01671 PMC4734579

[pbi12659-bib-0058] Meiri, D. , Tazat, K. , Cohen‐Peer, R. , Farchi‐Pisanty, O. , Aviezer‐Hagai, K. , Avni, A. and Breiman, A. (2010) Involvement of arabidopsis ROF2 (FKBP65) in thermotolerance. Plant Mol. Biol. 72, 191–203. 19876748 10.1007/s11103-009-9561-3

[pbi12659-bib-0059] Mishra, S.K. , Tripp, J. , Winkelhaus, S. , Tschiersch, B. , Theres, K. , Nover, L. and Scharf, K.D. (2002) In the complex family of heat stress transcription factors, HsfA1 has a unique role as master regulator of thermotolerance in tomato. Genes Dev. 16, 1555–1567. 12080093 10.1101/gad.228802 PMC186353

[pbi12659-bib-0060] Mittler, R. and Blumwald, E. (2010) Genetic engineering for modern agriculture: challenges and perspectives. Annu. Rev. Plant Biol. 61, 443–462. 20192746 10.1146/annurev-arplant-042809-112116

[pbi12659-bib-0061] Mogk, A. , Kummer, E. and Bukau, B. (2015) Cooperation of Hsp70 and Hsp100 chaperone machines in protein disaggregation. Front. Mol. Biosci. 2, 22. 26042222 10.3389/fmolb.2015.00022 PMC4436881

[pbi12659-bib-0062] Nakashima, K. , Fujita, Y. , Kanamori, N. , Katagiri, T. , Umezawa, T. , Kidokoro, S. , Maruyama, K. *et al*. (2009) Three arabidopsis SnRK2 protein kinases, SRK2D/SnRK2.2, SRK2E/SnRK2.6/OST1 and SRK2I/SnRK2.3, involved in ABA signaling are essential for the control of seed development and dormancy. Plant Cell Physiol. 50, 1345–1363. 19541597 10.1093/pcp/pcp083

[pbi12659-bib-0063] Neudegger, T. , Verghese, J. , Hayer‐Hartl, M. , Hartl, F.U. and Bracher, A. (2016) Structure of human heat‐shock transcription factor 1 in complex with DNA. Nat. Struct. Mol. Biol. 1, 140–146. 10.1038/nsmb.3149 26727489

[pbi12659-bib-0064] Niforou, K. , Cheimonidou, C. and Trougakos, I.P. (2014) Molecular chaperones and proteostasis regulation during redox imbalance. Redox. Biol. 1, 323–332. 10.1016/j.redox.2014.01.017 PMC3926111 24563850

[pbi12659-bib-0065] Nishizawa, A. , Yabuta, Y. , Yoshida, E. , Maruta, T. , Yoshimura, K. and Shigeoka, S. (2006) Arabidopsis heat shock transcription factor A2 as a key regulator in response to several types of environmental stress. Plant J. 48, 535–547. 17059409 10.1111/j.1365-313X.2006.02889.x

[pbi12659-bib-0066] Nishizawa‐Yokoi, A. , Nosaka, R. , Hayashi, H. , Tainaka, H. , Maruta, T. , Tamoi, M. , Ikeda, M. *et al*. (2011) HsfA1d and HsfA1e involved in the transcriptional regulation of Hsfa2 function as key regulators for the hsf signaling network in response to environmental stress. Plant Cell Physiol. 52, 933–945. 21471117 10.1093/pcp/pcr045

[pbi12659-bib-0067] Noël, L.D. , Cagna, G. , Stuttmann, J. , Wirthmüller, L. , Betsuyaku, S. , Witte, C.‐P. , Bhat, R. *et al*. (2007) Interaction between SGT1 and cytosolic/nuclear HSC70 chaperones regulates Arabidopsis immune responses. Plant Cell, 19, 4061–4076. 18065690 10.1105/tpc.107.051896 PMC2217652

[pbi12659-bib-0068] Nover, L. , Bharti, K. , Döring, P. , Mishra, S.K. , Ganguli, A. and Scharf, K.D. (2001) Arabidopsis and the heat stress transcription factor world: how many heat stress transcription factors do we need? Cell Stress Chaperones, 6, 177–189. 11599559 10.1379/1466-1268(2001)006<0177:aathst>2.0.co;2 PMC434399

[pbi12659-bib-0069] Ogawa, D. , Yamaguchi, K. and Nishiuchi, T. (2007) High‐level overexpression of the Arabidopsis HsfA2 gene confers not only increased themotolerance but also salt/osmotic stress tolerance and enhanced callus growth. J. Exp. Bot. 58, 3373–3383. 17890230 10.1093/jxb/erm184

[pbi12659-bib-0070] Oh, C.S. and Martin, G.B. (2011) Tomato 14‐3‐3 protein TFT7 interacts with a MAP kinase kinase to regulate immunity‐associated programmed cell death mediated by diverse disease resistance proteins. J. Biol. Chem. 286, 14129–14136. 21378171 10.1074/jbc.M111.225086 PMC3077614

[pbi12659-bib-0071] Park, C.‐J. and Seo, Y.‐S. (2015) Heat shock proteins: a review of the molecular chaperones for plant immunity. Plant Pathol J. 31, 323–333. 26676169 10.5423/PPJ.RW.08.2015.0150 PMC4677741

[pbi12659-bib-0072] Pérez‐Salamó, I. , Papdi, C. , Rigó, G. , Zsigmond, L. , Vilela, B. , Lumbreras, V. , Nagy, I. *et al*. (2014) The heat shock factor A4A confers salt tolerance and is regulated by oxidative stress and the mitogen‐activated protein kinases MPK3 and MPK6. Plant Physiol. 165, 319–334. 24676858 10.1104/pp.114.237891 PMC4012591

[pbi12659-bib-0073] Pulido, P. , Llamas, E. , Llorente, B. , Ventura, S. , Wright, L.P. and Rodríguez‐Concepción, M. (2016) Specific Hsp100 chaperones determine the fate of the first enzyme of the plastidial isoprenoid pathway for either refolding or degradation by the stromal clp protease in arabidopsis. PLoS Genet. 12, e1005824. 26815787 10.1371/journal.pgen.1005824 PMC4729485

[pbi12659-bib-0074] Qin, F. , Sakuma, Y. , Tran, L.‐S.P. , Maruyama, K. , Kidokoro, S. , Fujita, Y. , Fujita, M. *et al*. (2008) Arabidopsis DREB2A‐interacting proteins function as RING E3 ligases and negatively regulate plant drought stress‐responsive gene expression. Plant Cell, 20, 1693–1707. 18552202 10.1105/tpc.107.057380 PMC2483357

[pbi12659-bib-0075] Rasmussen, S. , Barah, P. , Suarez‐Rodriguez, M.C. , Bressendorff, S. , Friis, P. , Costantino, P. , Bones, A.M. *et al*. (2013) Transcriptome responses to combinations of stresses in Arabidopsis. Plant Physiol. 161, 1783–1794. 23447525 10.1104/pp.112.210773 PMC3613455

[pbi12659-bib-0076] Sakuma, Y. (2006a) Functional analysis of an arabidopsis transcription factor, DREB2A, involved in drought‐responsive gene expression. Plant Cell, 18, 1292–1309. 16617101 10.1105/tpc.105.035881 PMC1456870

[pbi12659-bib-0077] Sakuma, Y. , Maruyama, K. , Qin, F. , Osakabe, Y. , Shinozaki, K. and Yamaguchi‐Shinozaki, K. (2006b) Dual function of an Arabidopsis transcription factor DREB2A in water‐stress‐responsive and heat‐stress‐responsive gene expression. Proc. Natl Acad. Sci. USA, 103, 18822–18827. 17030801 10.1073/pnas.0605639103 PMC1693746

[pbi12659-bib-0078] Scharf, K.D. , Berberich, T. , Ebersberger, I. and Nover, L. (2012) The plant heat stress transcription factor (Hsf) family: structure, function and evolution. Biochim. Biophys. Acta, 1819, 104–119. 22033015 10.1016/j.bbagrm.2011.10.002

[pbi12659-bib-0079] Schramm, F. , Larkindale, J. , Kiehlmann, E. , Ganguli, A. , Englich, G. , Vierling, E. and Von Koskull‐Döring, P. (2008) A cascade of transcription factor DREB2A and heat stress transcription factor HsfA3 regulates the heat stress response of Arabidopsis. Plant J. 53, 264–274. 17999647 10.1111/j.1365-313X.2007.03334.x

[pbi12659-bib-0080] Shafikova, T.N. , Omelichkina, Y.V. , Soldatenko, A.S. , Enikeev, A.G. , Kopytina, T.V. , Rusaleva, T.M. and Volkova, O.D. (2013) Tobacco cell cultures transformed by the hsp101 gene exhibit an increased resistance to Clavibacter michiganensis ssp. sepedonicus. Dokl. Biol. Sci. 450, 165–167. 23821058 10.1134/S0012496613030162

[pbi12659-bib-0081] Shirasu, K. (2009) The HSP90‐SGT1 chaperone complex for NLR immune sensors. Annu. Rev. Plant Biol. 60, 139–164. 19014346 10.1146/annurev.arplant.59.032607.092906

[pbi12659-bib-0082] Singh, D. and Laxmi, A. (2015) Transcriptional regulation of drought response: a tortuous network of transcriptional factors. Front. Plant Sci. 6, 895. 26579147 10.3389/fpls.2015.00895 PMC4625044

[pbi12659-bib-0083] Sjögren, L.L.E. , MacDonald, T.M. , Sutinen, S. and Clarke, A.K. (2004) Inactivation of the clpC1 gene encoding a chloroplast Hsp100 molecular chaperone causes growth retardation, leaf chlorosis, lower photosynthetic activity, and a specific reduction in photosystem content. Plant Physiol. 136, 4114–4126. 15563614 10.1104/pp.104.053835 PMC535842

[pbi12659-bib-0084] Sugio, A. , Dreos, R. , Aparicio, F. and Maule, A.J. (2009) The cytosolic protein response as a subcomponent of the wider heat shock response in Arabidopsis. Plant Cell. 21, 642–654. 19244141 10.1105/tpc.108.062596 PMC2660624

[pbi12659-bib-0085] Suzuki, N. , Rivero, R.M. , Shulaev, V. , Blumwald, E. and Mittler, R. (2014) Abiotic and biotic stress combinations. New Phytol. 203, 32–43. 24720847 10.1111/nph.12797

[pbi12659-bib-0086] Swindell, W.R. , Huebner, M. and Weber, A.P. (2007) Transcriptional profiling of Arabidopsis heat shock proteins and transcription factors reveals extensive overlap between heat and non‐heat stress response pathways. BMC Genom. 8, 125. 10.1186/1471-2164-8-125 PMC1887538 17519032

[pbi12659-bib-0087] Taipale, M. , Krykbaeva, I. , Koeva, M. , Kayatekin, C. , Westover, K.D. , Karras, G.I. and Lindquist, S. (2012) Quantitative analysis of Hsp90‐client interactions reveals principles of substrate recognition. Cell, 150, 987–1001. 22939624 10.1016/j.cell.2012.06.047 PMC3894786

[pbi12659-bib-0088] Takahashi, A. , Casais, C. , Ichimura, K. and Shirasu, K. (2003) HSP90 interacts with RAR1 and SGT1 and is essential for RPS2‐mediated disease resistance in Arabidopsis. Proc. Natl Acad. Sci. USA, 100, 11777–11782. 14504384 10.1073/pnas.2033934100 PMC208834

[pbi12659-bib-0089] Taki, N. , Sasaki‐Sekimoto, Y. , Obayashi, T. , Kikuta, A. , Kobayashi, K. , Ainai, T. , Yagi, K. *et al*. (2005) 12‐Oxo‐phytodienoic acid triggers expression of a distinct set of genes and plays a role in wound‐induced gene expression in arabidopsis. Plant Physiol. 139, 1268–1283. 16258017 10.1104/pp.105.067058 PMC1283764

[pbi12659-bib-0090] Tang, T. , Yu, A. , Li, P. , Yang, H. , Liu, G. and Liu, L. (2016) Sequence analysis of the Hsp70 family in moss and evaluation of their functions in abiotic stress responses. Sci. Rep. 6, 33650. 27644410 10.1038/srep33650 PMC5028893

[pbi12659-bib-0091] Volkov, R.A. , Panchuk, I.I. , Mullineaux, P.M. and Schöffl, F. (2006) Heat stress‐induced H_2_O_2_ is required for effective expression of heat shock genes in Arabidopsis. Plant Mol. Biol. 61, 733–746. 16897488 10.1007/s11103-006-0045-4

[pbi12659-bib-0092] Waddington, C.H. (1961) Genetic assimilation. Adv. Genet. 10, 257–293. 14004267 10.1016/s0065-2660(08)60119-4

[pbi12659-bib-0093] Walter, P. and Ron, D. (2012) The unfolded protein response: from stress pathway to homeostatic regulation. Science, 334, 1081–1086. 10.1126/science.1209038 22116877

[pbi12659-bib-0094] Wang, P. , Xue, L. , Batelli, G. , Lee, S. , Hou, Y.‐J. , Van Oosten, M.J. , Zhang, H. *et al*. (2013) Quantitative phosphoproteomics identifies SnRK2 protein kinase substrates and reveals the effectors of abscisic acid action. Proc. Natl Acad. Sci. USA, 110, 11205–11210. 23776212 10.1073/pnas.1308974110 PMC3703982

[pbi12659-bib-0095] Wells, D.R. , Tanguay, R.L. , Le, H. and Gallie, D.R. (1998) HSP101 functions as a specific translational regulatory protein whose activity is regulated by nutrient status. Genes Dev. 12, 3236–3251. 9784498 10.1101/gad.12.20.3236 PMC317219

[pbi12659-bib-0096] Winter, D. , Vinegar, B. , Nahal, H. , Ammar, R. , Wilson, G.V. and Provart, N.J. (2007) An “electronic fluorescent pictograph” Browser for exploring and analyzing large‐scale biological data sets. PLoS ONE, 2, e718. 17684564 10.1371/journal.pone.0000718 PMC1934936

[pbi12659-bib-0097] Wu, T.‐Y. , Juan, Y.‐T. , Hsu, Y.‐H. , Wu, S.‐H. , Liao, H.‐T. , Fung, R.W.M. and Charng, Y.‐Y. (2013) Interplay between heat shock proteins HSP101 and HSA32 prolongs heat acclimation memory posttranscriptionally in Arabidopsis. Plant Physiol. 161, 2075–2084. 23439916 10.1104/pp.112.212589 PMC3613477

[pbi12659-bib-0098] Xue, G.P. , Drenth, J. and McIntyre, C.L. (2015) TaHsfA6f is a transcriptional activator that regulates a suite of heat stress protection genes in wheat (Triticum aestivum L.) including previously unknown Hsf targets. J. Exp. Bot. 66, 1025–1039. 25428996 10.1093/jxb/eru462 PMC4321556

[pbi12659-bib-0099] Yokotani, N. , Ichikawa, T. , Kondou, Y. , Matsui, M. , Hirochika, H. , Iwabuchi, M. and Oda, K. (2008) Expression of rice heat stress transcription factor OsHsfA2e enhances tolerance to environmental stresses in transgenic Arabidopsis. Planta, 227, 957–967. 18064488 10.1007/s00425-007-0670-4

[pbi12659-bib-0100] Yoshida, T. , Sakuma, Y. , Todaka, D. , Maruyama, K. , Qin, F. , Mizoi, J. , Kidokoro, S. *et al*. (2008) Functional analysis of an Arabidopsis heat‐shock transcription factor HsfA3 in the transcriptional cascade downstream of the DREB2A stress‐regulatory system. Biochem. Biophys. Res. Commun. 368, 515–521. 18261981 10.1016/j.bbrc.2008.01.134

[pbi12659-bib-0101] Yoshida, T. , Ohama, N. , Nakajima, J. , Kidokoro, S. , Mizoi, J. , Nakashima, K. , Maruyama, K. *et al*. (2011) Arabidopsis HsfA1 transcription factors function as the main positive regulators in heat shock‐responsive gene expression. Mol. Genet. Genom. 286, 321–332. 10.1007/s00438-011-0647-7 21931939

[pbi12659-bib-0102] Yu, A. , Li, P. , Tang, T. , Wang, J. , Chen, Y. and Liu, L. (2015) Roles of Hsp70s in stress responses of microorganisms, plants, and animals. Biomed Res. Int. 2015, 510319. 26649306 10.1155/2015/510319 PMC4663327

[pbi12659-bib-0103] Zhang, Y. , Dorey, S. , Swiderski, M. and Jones, J.D.G. (2004) Expression of RPS4 in tobacco induces an AvrRps4‐independent HR that requires EDS1, SGT1 and HSP90. Plant J. 40, 213–224. 15447648 10.1111/j.1365-313X.2004.02201.x

[pbi12659-bib-0104] Zhang, X.‐C. , Millet, Y.A. , Cheng, Z. , Bush, J. and Ausubel, F.M. (2015) Jasmonate signalling in Arabidopsis involves SGT1b–HSP70–HSP90 chaperone complexes. Nature Plants, 1, 15049. 27054042 10.1038/nplants.2015.49 PMC4819967

[pbi12659-bib-0105] Zhang, H. , Li, L. , Ye, T. , Chen, R. , Gao, X. and Xu, Z. (2016) Molecular characterization, expression pattern and function analysis of theOsHSP90family in rice. Biotechnol. Biotechnol. Equip. 30, 669–676.

[pbi12659-bib-0106] Zhou, L. , Liu, Z. , Liu, Y. , Kong, D. , Li, T. , Yu, S. , Mei, H. *et al*. (2016) A novel gene OsAHL1 improves both drought avoidance and drought tolerance in rice. Sci. Rep. 6, 30264. 27453463 10.1038/srep30264 PMC4958981

[pbi12659-bib-0107] Zhu, B. , Chunjiang, B. , bullet, Y. , Lü, H. , Xiaojun, B. , bullet, C. , Chai, G. *et al*. (2006) Identification and characterization of a novel heat shock transcription factor gene, GmHsfA1, in soybeans (Glycine max). J. Plant. Res. 119, 247–256. 16570125 10.1007/s10265-006-0267-1

